# Analysis of the Transcriptome in *Aspergillus tamarii* During Enzymatic Degradation of Sugarcane Bagasse

**DOI:** 10.3389/fbioe.2018.00123

**Published:** 2018-09-18

**Authors:** Glaucia Emy Okida Midorikawa, Camila Louly Correa, Eliane Ferreira Noronha, Edivaldo Ximenes Ferreira Filho, Roberto Coiti Togawa, Marcos Mota do Carmo Costa, Orzenil Bonfim Silva-Junior, Priscila Grynberg, Robert Neil Gerard Miller

**Affiliations:** ^1^Departamento de Biologia Celular, Universidade de Brasília, Brasília, Brazil; ^2^Embrapa Recursos Genéticos e Biotecnologia, Parque Estação Biológica, Brasília, Brazil

**Keywords:** *Aspergillus tamarii*, transcriptome, carbohydrate-active enzymes, XlnR, ClrA, sugar transporters, lignocellulose, bioethanol

## Abstract

The production of bioethanol from non-food agricultural residues represents an alternative energy source to fossil fuels for incorporation into the world's economy. Within the context of bioconversion of plant biomass into renewable energy using improved enzymatic cocktails, Illumina RNA-seq transcriptome profiling was conducted on a strain of *Aspergillus tamarii*, efficient in biomass polysaccharide degradation, in order to identify genes encoding proteins involved in plant biomass saccharification. Enzyme production and gene expression was compared following growth in liquid and semi-solid culture with steam-exploded sugarcane bagasse (SB) (1% *w/v*) and glucose (1% *w/v*) employed as contrasting sole carbon sources. Enzyme production following growth in liquid minimum medium supplemented with SB resulted in 0.626 and 0.711 UI.mL^−1^ xylanases after 24 and 48 h incubation, respectively. Transcriptome profiling revealed expression of over 7120 genes, with groups of genes modulated according to solid or semi-solid culture, as well as according to carbon source. Gene ontology analysis of genes expressed following SB hydrolysis revealed enrichment in xyloglucan metabolic process and xylan, pectin and glucan catabolic process, indicating up-regulation of genes involved in xylanase secretion. According to carbohydrate-active enzyme (CAZy) classification, 209 CAZyme-encoding genes were identified with significant differential expression on liquid or semi-solid SB, in comparison to equivalent growth on glucose as carbon source. Up-regulated CAZyme-encoding genes related to cellulases (CelA, CelB, CelC, CelD) and hemicellulases (XynG1, XynG2, XynF1, XylA, AxeA, arabinofuranosidase) showed up to a 10-fold log2FoldChange in expression levels. Five genes from the AA9 (GH61) family, related to lytic polysaccharide monooxygenase (LPMO), were also identified with significant expression up-regulation. The transcription factor gene XlnR, involved in induction of hemicellulases, showed up-regulation on liquid and semi-solid SB culture. Similarly, the gene ClrA, responsible for regulation of cellulases, showed increased expression on liquid SB culture. Over 150 potential transporter genes were also identified with increased expression on liquid and semi-solid SB culture. This first comprehensive analysis of the transcriptome of *A. tamarii* contributes to our understanding of genes and regulatory systems involved in cellulose and hemicellulose degradation in this fungus, offering potential for application in improved enzymatic cocktail development for plant biomass degradation in biorefinery applications.

## Introduction

The production of renewable energy is one of the greatest challenges of the twenty-first Century. Whilst dependency upon fossil fuels is associated with depleting oil reserves and greenhouse gas emissions, plant biomass, by contrast, with its' global abundancy, represents a sustainable and environmentally clean energy source (Goldemberg, 2007; Tan et al., 2016).

The production of bioethanol from non-food agricultural residues such as lignocellulosic trash, grasses and woods, is known as second-generation (2G) ethanol (Alvira et al., 2010), and is considered a promising alternative energy source to fossil fuels for incorporation into the world's economy. Brazil is currently one of the principal agricultural producers, as an important supplier of both food and industrial crops. Sugarcane is planted over an area of almost 9 million hectares, with an annual production of over 620 million tons (CONAB, 2018). Whilst around 45% of the crop production is employed for sugar extraction, the majority is used in the bioethanol industry, with estimates of production of 28 billion liters of anhydrous and hydrated ethanol for 2018/2019 (CONAB, 2018). Bioethanol production in Brazil is based almost exclusively on first-generation technologies, whereby the sucrose content of the plant is converted into ethanol. In this process, sugarcane bagasse will accumulate as an agricultural residue (Goldemberg, 2008). Whilst the burning of bagasse currently serves as an energy source in bioethanol mills, as this biomass represents approximately one-third of the energy content of the crop, the conversion of the lignocellulose component of the cell wall into fermentable hexose (glucose) and pentose (e.g., D-xylose and L-arabinose) sugars offers considerable potential for increased 2G ethanol production, potentially by up to 40% (Amorim et al., 2011). Two Brazilian cellulosic ethanol plants came into operation in 2014, with capacities planned for production of up to 1 billion liters of ethanol per year from bagasse (Silva et al., 2017).

For economically viable 2G ethanol production, complete hydrolysis, or saccharification, of plant biomass is required. Such plant material is composed mainly of polysaccharide crystalline microfibers of cellulose (40–50%), followed in abundance by a matrix of various hemicelluloses and pectins (25–35%), in addition to the polyaromatic lignin (15–20%) (Lin and Tanaka, 2006; Ragauskas et al., 2006; Jordan et al., 2012; Guerriero et al., 2016). Efficient biorefinary-based conversion of this material is hampered due to the recalcitrance of lignocelluloses (Chundawat et al., 2011). In the case of sugarcane bagasse, lignocellulose sugars vary in terms of identity and branching, comprising residues of glucose (60%), xylose (13%), arabinose (6%), mannose (3%), galactose (1,5%), and less than 1% fructose and rhamnose (Häkkinen et al., 2012).

As cellulases and hemicellulases remain costly, increasing the costs of 2G bioethanol production, a continued characterization of sources of such enzymes, together with an improved understanding of the mechanisms involved in enzyme secretion and enzyme efficiency are of fundamental importance for the biofuel industry. Hydrolytic enzymes appropriate for fermentation of available sugars in lignocelluloses are known to be secreted by a wide variety of bacteria and filamentous fungi, with the latter often producing not only a diverse array of extracellular lignocellulolitic enzymes, but also displaying efficiency in secretion of such enzymes in high quantities (Phitsuwan et al., 2013). For this reason, fungi are today the principal source of hydrolytic enzymes for this industrial application (Sims et al., 2010; Couturier et al., 2012).

Lignocellulolytic fungi typically produce extensive sets of carbohydrate-active enzymes (CAZymes) that correlate with their geographical origin habitat (Van Den Brink and De Vries, 2011). A number of ascomycete fungi, notably species members of the genera *Trichoderma* and *Aspergillus*, produce a range of cellulases and hemicellulases, which are today applied across numerous relevant industries for production of food, feed, paper, textiles and pharmaceuticals (Archer, 2000; de Souza et al., 2011). Whilst *Aspergillus niger* and *Trichoderma reseei* are currently employed in the production of commercial enzymatic cocktails for lignocellulosic biomass deconstruction (Singhania, 2011; Mohanram et al., 2013), the identification of additional sources of carbohydrate active enzymes will likely increase efficiency in the deconstruction of this biomass. As such, additional species have recently been screened as potential sources of cellulases, hemicellulases and accessory proteins for optimized industrial enzyme production (Brown et al., 2016; Cong et al., 2017; de Gouvêa et al., 2018).

The availability of whole genome sequences for fungi has improved understanding of fungal biodiversity with respect to plant cell wall degradation. *Aspergillus nidulans*, considered the model species of the genus given its' well-elucidated sexual cycle, possesses a genome sequence of 30.06 MB, with 9396 predicted genes (Galagan et al., 2005). Other characterized Aspergillus species of importance for the food, textile, pulp and paper industries, and potentially in 2G ethanol production, include *A. oryzae* and *A. niger. A. oryzae* has a total genome size of 37.12 MB, with 12336 predicted genes (Machida et al., 2005). Similarly, *A. niger* possesses a genome of 37.2 MB, with 14600 predicted genes (Pel et al., 2007). Comparison of gene sequences against the Carbohydrate-Active Enzymes Database (http://www.cazy.org/) (Cantarel et al., 2009) has revealed 186 genes related to polysaccharide hydrolysis in *A. nidulans*, 217 in *A. oryzae* and 171 in *A. niger* (Delmas et al., 2012).

In addition to gene discovery, the annotated genome sequences for these species serve as resources for analysis of the transcriptome in additional *Aspergillus* species without available genome sequences. Such analysis of transcriptional regulation of genes encoding hydrolytic enzymes in *Aspergillus* has been studied in relation to growth on different sugar carbon sources (Andersen et al., 2008; Jørgensen et al., 2009; Salazar et al., 2009). In relation to fermentation of sugarcane bagasse, microarray analysis provided information on gene expression modulation in *A. niger* (Guillemette et al., 2007; de Souza et al., 2011), with cellulases, hemicellulases and transporters identified with increased expression during growth on sugarcane bagasse in comparison to fructose. Subsequent RNAseq analysis of the *A. niger* transcriptome, following growth on wheat straw compared to simple sugars, revealed a CAZy gene representation change from 3% of total mRNA on 1% glucose to 19% on wheat straw, representing numerous enzymes from the classes of Glycoside Hydrolases (GH), Carbohydrate Esterases (CE), and Polysaccharide Lyases (PL) (Delmas et al., 2012). Further RNAseq-based analysis of gene expression in Aspergillus species following growth on sugarcane bagasse as carbon source has also revealed important information regarding regulatory mechanisms and genes encoding plant cell wall degrading enzymes, accessory proteins and transporters (Pullan et al., 2014; Brown et al., 2016; Borin et al., 2017; Cong et al., 2017; de Gouvêa et al., 2018).

The continued characterization of hydrolases, accessory proteins and the regulation of their expression in Aspergillus species that display efficiency in degradation of lignocellulose will further our understanding of their roles in saccharification. Given the importance of *Aspergillus tamarii* as an efficient producer of enzymes such as xylanases (El-Gindy et al., 2015; Monclaro et al., 2016), we utilized an Illumina RNA-seq approach to analyze the transcriptome in this fungus following semi-solid and liquid cultivation on steam-exploded bagasse (SB) compared gene expression following growth on glucose (G). Genes encoding cellulases and hemicellulases, transcription factors and transporters are characterized in relation to their differential expression following fungal growth on each carbon source. Data will benefit the development of improved fungal strains with increased ability to deconstruct lignocellulose and generate value-added bioproducts.

## Materials and methods

### Strain and culture conditions

A stock culture of a strain of *A. tamarii*, code BLU37, was provided by the fungal culture collection at the Enzymology Laboratory, University of Brasilia, Brazil (genetic heritage number 010237/2015-1). The strain was originally isolated into pure culture from natural composting cotton textile waste material in the Vale do Itajaí, Santa Catarina, Brazil (Siqueira et al., 2009) and maintained in the culture collection at −80°C in 50% glycerol.

Species identity reconfirmation was conducted by sequence analysis of the nuclear ribosomal DNA (rDNA) ITS1-5.8S-ITS2 region, together with specific regions of the β-tubulin and calmodulin genes. Genomic DNA was extracted according to Raeder and Broda (1985) from a 3 day old liquid culture in Czapek Yeast Extract medium (CYA) (Pitt and Hocking, 2009) incubated on an orbital shaker at 28°C. Each PCR reaction contained 10 ng genomic DNA, 2,5 mmol^−1^ of each primer, 1 mmol^−1^ dNTPs, 4 mmol^−1^ MgCl_2_, 1U of Taq Platinum^®^ polymerase (Invitrogen) and 1 x Taq Platinum® polymerase buffer (Invitrogen). Ribosomal DNA ITS regions were amplified using primers ITS5 and ITS4 (White et al., 1990), a β-tubulin gene region with primers Bt_2_a and Bt_2_b (Glass and Donaldson, 1995), and a calmodulin gene region amplified with primers Cmd5 and Cmd6 (Hong et al., 2006). PCR cycling was performed with the following programs: initial denaturation at 94°C for 4 min, 30 cycles of denaturation at 94°C for 1 min, primer annealing for 1 min, at 50°C for primers ITS5 and ITS4, and at 60°C for primers Bt2a, Bt2b, Cmd5 and Cmd6, extension at 72°C for 1 min, and a final extension period at 72°C for 5 min. PCR products were purified using ExoSAP-IT® (USB, Cleveland, Ohio, USA) and sequenced using Big Dye^®^ Terminator v3.1 Cycle Sequencing chemistry (Applied Biosystems, Foster City, CA, USA) on an ABI 3700 DNA sequencer (Applied Biosystems, Foster City, CA, USA). For molecular identification, sequences were compared against the nucleotide database NCBI using the BLASTn algorithm (Altschul et al., 1990). Ribosomal DNA ITS, β-tubulin and calmodulin gene sequences were deposited in GenBank under accession numbers MH540359, MH544272 and MH544273, respectively.

For analysis of gene expression in BLU37 following exposure to SB or glucose as carbon source, the strain was grown in either liquid or semi-solid minimal medium (KH_2_PO_4_ 7 g; K_2_HPO_4_ 2 g; MgSO_4_ 0.4 g; (NH_4_)_2_SO_4_ 1.6 g, pH 7.0, per liter of distilled water), containing SB (1% *w/v*) or glucose (1% *w/v*) (Sigma Aldrich) as exclusive carbon source. In order to guarantee elimination of reducing sugars, prior to fungal inoculation, SB was repeatedly washed with deionized water until reducing sugars were no longer detectable by the colorimetric dinitrosalicylic acid (DNS) assay (Miller, 1959). Liquid cultures were grown in 100 mL of media in Erlenmeyer flasks, whilst semi-solid cultures were grown on petri plates with media supplemented with agar (15 g L^−1^). Fungal spores at a concentration of 1 × 10^8^ conidiospores mL^−1^ were used as inocula, with cultures then incubated at 28°C and 150 rpm for 36 and 48 h. Fungal cultures were arranged in a randomized block design, with three replicates for each treatment and time point. Growth treatments were labeled as follows: liquid medium, SB carbon source, 36 h incubation (LB36); liquid medium, glucose carbon source, 36 h incubation (LG36); liquid medium, SB carbon source, 48 h incubation (LB48); liquid medium, glucose carbon source, 48 h incubation (LG48); semi-solid medium SB carbon source, 36 h incubation (SB36); semi-solid medium, glucose carbon source, 36 h incubation (SG36); semi-solid medium, SB carbon source, 48 h incubation (SB48); semi-solid medium, glucose carbon source, 48 h incubation (SG48).

### Analysis of enzymatic activities

Analysis of hydrolytic enzyme secretion was conducted following fungal growth in the liquid minimal medium with SB (1% *w/v*) or glucose (1% *w/v*) as carbon source. Enzyme activities were evaluated over a 10 day period at 24 h intervals. Xylanase, CMCase, pectinase and FPase assays were determined using the DNS assay at pH 5.0. Each assay comprised 10 μl of the fungal secretome, together with xylan (1% *w/v*), carboxy methylcellulose (1% *w/v*), or pectin (1% *w/v*) as substrate. An assay for FPase activity, as a measurement of total cellulases, was conducted according to Ghose (1987). All assays were conducted with at least three replicates. Quantification of reduced sugars from the assays was conducted using a spectrophotometer at an absorbance of 540 nm (Spectra Max), calibrated using standard curves of glucose, xylose and galacturonic acid. Absorbance values for each assay were calculated in international units (UI), where 1 UI was defined as the amount of enzyme necessary to release 1 μmol of reducing sugars per minute per liter by hydrolysis of each crude substrate.

### Vizualization of fungal colonization of SB by scanning electron microscopy

Following 36 and 48 h mycelial growth, liquid minimum medium culture supplemented with SB was filtered with Whatman^®^ filter paper n°1 and washed with Karnovsky buffer (0.05M; pH 7.2). Samples were fixed for 4 h in a fresh solution of 0.05M cacodylate buffer at pH 7.4. Following dehydration with acetone, samples were postfixed for 1 h using 1% osmium tetroxide. Samples were washed in liquid CO_2_ at 4°C, dried in a critical point drier (Emitech K850, Kent, UK), mounted on copper stubs then sputter coated with 20 nm gold particles. Prepared samples were observed under scanning electron microscopy using a Zeiss DSM 962 scanning electron microscope.

### Total RNA extraction and illumina RNA-seq

Following 36 and 48 h incubation, total RNA was extracted from each culture according to a standard phenol/chloroform method (Brasileiro et al., 2015). Mycelia from liquid media was collected by filtration with Whatman® filter paper n°1, whilst from semi-solid cultures mycelia from the surface of each agar plate was collected manually using a sterilized spatula. Extracted total RNA was quantified and integrity determined using an Agilent 2100 Bioanalyzer and RNA LabChip® kit system (Agilent Technologies, Santa Clara, CA, USA). Isolation of mRNA, cDNA library construction and Illumina RNA-seq were conducted by Eurofins MWG Operon (Louisville, KY, USA). All treatments from the replicate bioassays were paired-end sequenced (2 × 100 bases) using TruSeq RNA Chemistry v3 on two flow cell channels of an Illumina Hiseq2000 system (Illumina Inc., San Diego CA, USA).

### Bioinformatics analysis

#### Read mapping and assembly

Quality was determined for sequence reads from each cDNA library using ea-utils (Aronesty, 2011). For assignment of reads to gene models for the genus, high quality sequences (Fastq QC > 30) were mapped against the reference annotated genome sequence for the phylogenetically related species *A. oryzae*, strain RIB40 (National Research Institute of Brewing Stock Culture and ATCC-42149 (Machida et al., 2005), publically available at DOGAN (http://www.bio.nite.go.jp/dogan/Top). Alignment and assembly were conducted using the programs TopHat (Trapnell et al., 2009) and Cufflinks (Trapnell et al., 2010).

#### Analysis of gene expression levels for normalized data

Genes with statistically significant differential expression between the evaluated growth conditions were identified on the basis of comparisons of *in silico* data. Read counts aligned to each gene were determined using the Python script HTseq-count (Anders et al., 2015). Differences in gene expression levels between treatments were calculated using the DEGseq program (Wang et al., 2010a). Differentially expressed genes (DEGs) between evaluated treatments were considered to be significant if a log2 fold change (FC) was at least ≥2-fold and at a probability level of *p* ≤ 0.01.

#### Gene ontology and analysis of enrichment

Using the program FUNC (Prüfer et al., 2007), a hypergeometric test enabled analysis of both over- and under-representation of DEGs according to gene function classification within gene ontology categories (GO). Redundancy in category terms was eliminated using the program REVIGO (http://revigo.irb.hr/).

#### Carbohydrate-active enzymes

Identification of genes encoding hydrolytic enzymes was conducted through alignment against the Carbohydrate-Active Enzymes (CAZymes) database (http://www.cazy.org/) (Cantarel et al., 2009), to enable classification of glycosyl hydrolases, glycosyltransfereases, carbohydrate-binding modules and carbohydrate esterases.

#### Transcription factors

Transcription factors were identified following alignment of gene sequences against the Fungal Transcription Factor Database (FTFD) (http://ftfd.snu.ac.kr/index.php?a=view).

### Validation of RNAseq-derived DEGs by RT-qPCR

To validate DEGs identified on the basis of *in silico* transcriptome data, a RT-qPCR analysis was conducted according to MIQE guidelines (Bustin et al., 2009). All specific primers were designed with Primer Express® software (Applied Biosystems) and evaluated at OligoAnalyzer 3.1 – IDT (https://www.idtdna.com/calc/analyzer). All cDNA libraries were synthesized using the same RNA samples employed for RNAseq analysis. Three independent biological replicates were analyzed for each carbon source and time point treatment, with three technical replicates per amplification. Total RNA was treated with 2U of Amplification Grade DNase I (Invitrogen, Carlsbad, CA, USA) and cDNA was synthesized with Oligo(dT)20 primers (Invitrogen, Carlsbad, CA, USA) and SuperScript® II Reverse Transcriptase (Invitrogen, Carlsbad, CA, USA). PCR was carried on a Step One Plus Real Time PCR System (Applied Biosystems) using a Platinum® SYBR® Green qPCR Super Mix-UDG w/ROX kit (Invitrogen, Carlsbad, CA, USA) according to the manufacturer's recommendations and 1 μL of template cDNA. Thermocycling was performed with 40 cycles of denaturation at 95°C for 15 s, followed by primer annealing and extension at 60°C for 30 s. GAPDH (Wang et al., 2010b) and β-tubulin (Mckelvey and Murphy, 2010) were employed as stable reference genes. Cycle threshold (Ct) values were determined using the program SDS 2.2.2 (Applied Biosystems, Foster City, USA), with specificity of PCR products for each primer set verified according to the Tm (dissociation) of the amplified products. Individual amplification efficiencies were calculated with the program LinRegPCR, version 2013.0, using a window-of-linearity. Gene expression values were calculated according to the 2-ΔΔCT method (Livak and Schmittgen, 2001).

## Results

### Fungal growth on steam-exploded sugarcane bagasse

**Figure 1:**
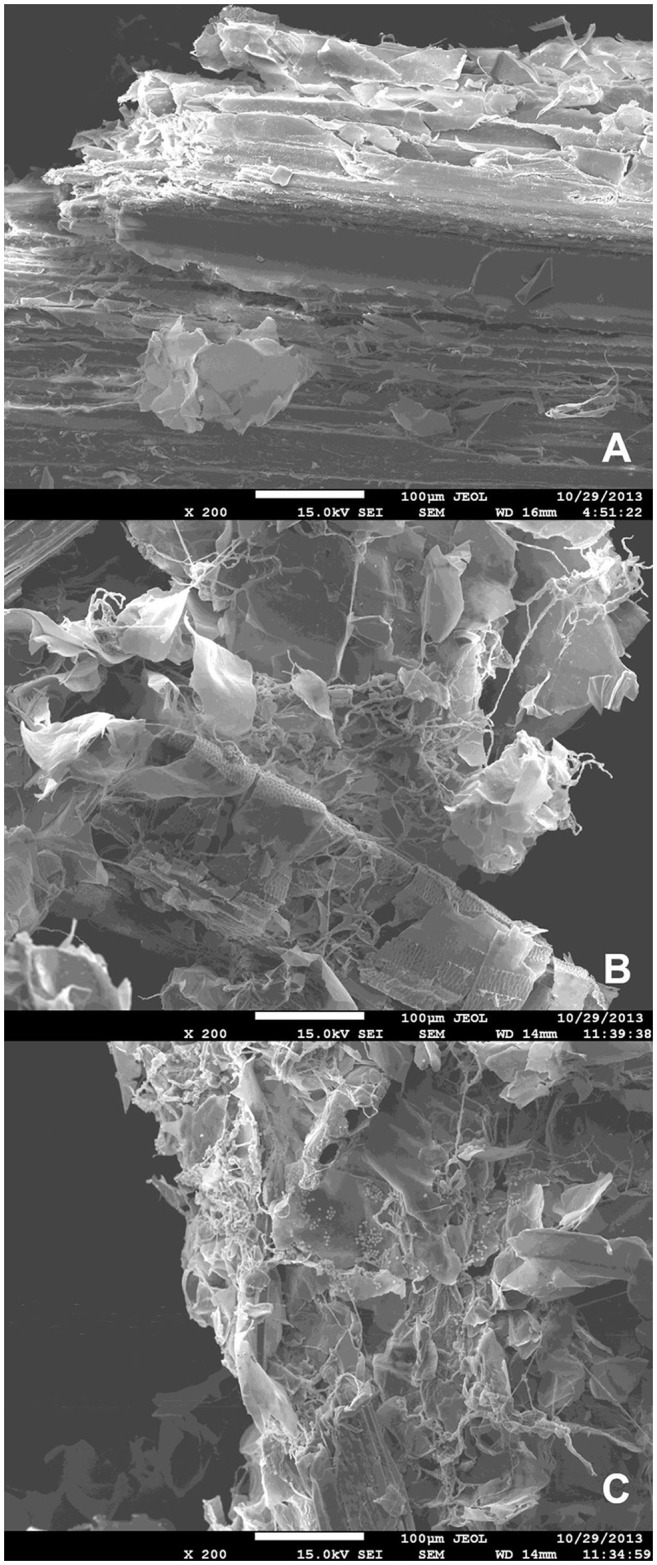
Scanning electron microscopy images of **(A)** non-inoculated steam-exploded sugarcane bagasse parenchyma in liquid minimal medium culture; **(B)** steam-exploded sugarcane bagasse parenchyma in liquid minimal medium culture following 36 h incubation with *A. tamarii* BLU37; **(C)** steam-exploded sugarcane bagasse parenchyma in liquid minimal medium culture following 48 h incubation with *A. tamarii* BLU37. Bars = 100 μm; 200X magnification.

Scanning electron microscopy-based observation of liquid minimal medium culture supplemented with SB following inoculation with *A. tamarii* conidia revealed evidence for degradation of sugarcane bagasse after incubation with the fungus (Figure 1). Homogeneous SB parenchyma fragments prior to inoculation can be observed in Figure 1A, with mycelial growth and resultant disruption of the parenchyma surface clearly visible in Figures 1B,C, following 36 and 48 h fungal colonization.

### Evaluation of enzyme production

**Figure 2:**
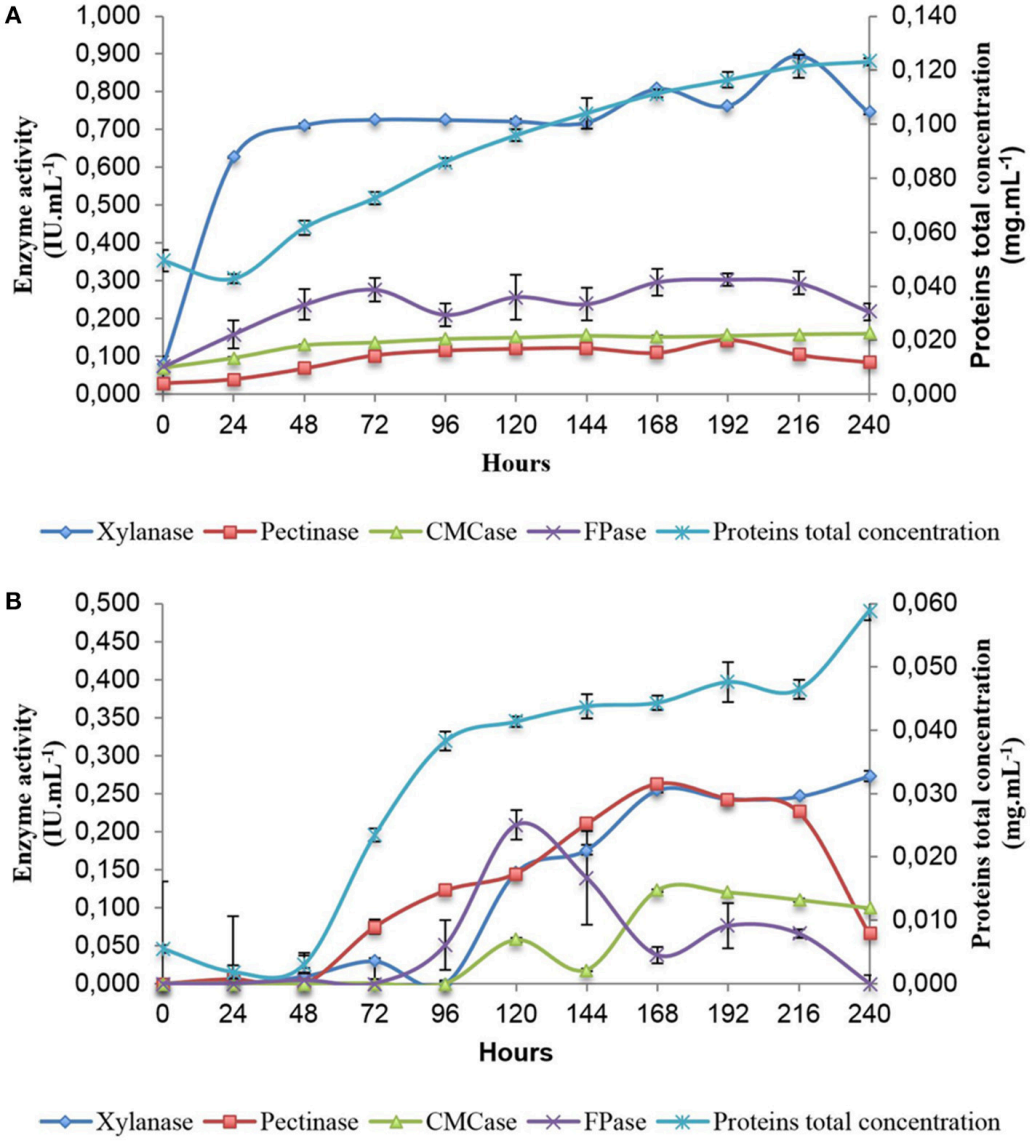
Enzymatic activity profile of A. *tamarii* BLU37 in response to xylan, pectin, carboxymethylcellulose and filter paper substrates following growth in liquid minimal medium supplemented with sugarcane (1% *w/v*) **(A)** or glucose (1% *w/v*) **(B)** as carbon source. Activities were determined through a DNS assay at pH 5.0.

In order to gain an understanding of the genes and pathways involved in xylanase and cellulase induction following exposure to SB, analysis of enzyme secretion in *A. tamarii* BLU37 was firstly conducted over a 10 day period following growth on liquid minimal medium culture supplemented with SB, as well as on the same medium with a glucose carbon source as control (Figure 2). Xylanase activity was shown to rapidly increase during the first 24 h on SB (0.626 ± 0.001), in contrast to growth on glucose (0.000 ± 0.004), with activity remaining relatively constant over the 10 day period. The activities of CMCases, pectinases and FPases at 24 h on SB were inferior in comparison to xylanases (0.096 ± 0.002, 0.039 ± 0.004, and 0.159 ± 0.037, respectively), although they were clearly induced by SB when compared to values for these enzymes following growth in glucose (0.000 ± 0.002, 0.006 ± 0.007, and 0.000 ± 0.088, respectively). Again, the expression of these enzymes remained relatively constant throughout the 10 day evaluation time course. Given the kinetics of xylanase production on SB, together with evidence for carbon catabolic repression during the first 48 h of growth on glucose, 36 and 48 h were selected as time points for analysis of the transcriptome of *A. tamarii* BLU37 in response to SB or glucose as carbon source.

### Transcriptome of *aspergillus tamarii* BLU37 in response to sugarcane bagasse

#### Sequence metrics

Illumina Hiseq2000-sequenced cDNA libraries resulted in a total of 634,255,527 reads with lengths between 75 and 85 bp, totaling 83.82 Gb of raw data. Illumina quality filtering indicated no information loss on the basis of pass-filter data. High quality sequences (Fastq QC>30) averaged 80.34% after adapter trimming (Supplementary Table 1). The total of paired and unpaired reads mapping to the reference genome *A. oryzae* RIB40 was 94 and 84%, respectively. An indel percentage of approximately 0.002% was observed in quality filtered sequences, with a low total of chimeric reads, at 0.000949%. All Illumina RNAseq data was deposited at the NCBI Sequence Read Archive (SRA) database (BioProject ID PRJNA479954, SRA accession: SRP152413).

#### Gene expression modulation

Sequence data generated from each of the cDNA libraries successfully aligned to over 7120 of the 12074 gene models in the reference *A. oryzae* RIB40 genome. Such homogeneity in characterized genes across cDNA libraries indicates a high quality and coverage of sequenced mRNA.

Analysis of fold change in gene expression was conducted on read counts aligned to each gene model in the reference *A. oryzae* RIB40 genome. DEGs with significant fold change (at least ≥2-fold and at a probability level of *p* ≤ 0.01) between treatments were identified through comparison of mapped read counts for genes expressed in cultures grown on SB as carbon source, in contrast read counts following growth on respective glucose controls. Differential gene expression analysis was also conducted in relation to culture format (liquid or semi-solid) and growth period (36 or 48 h).

**Figure 3:**
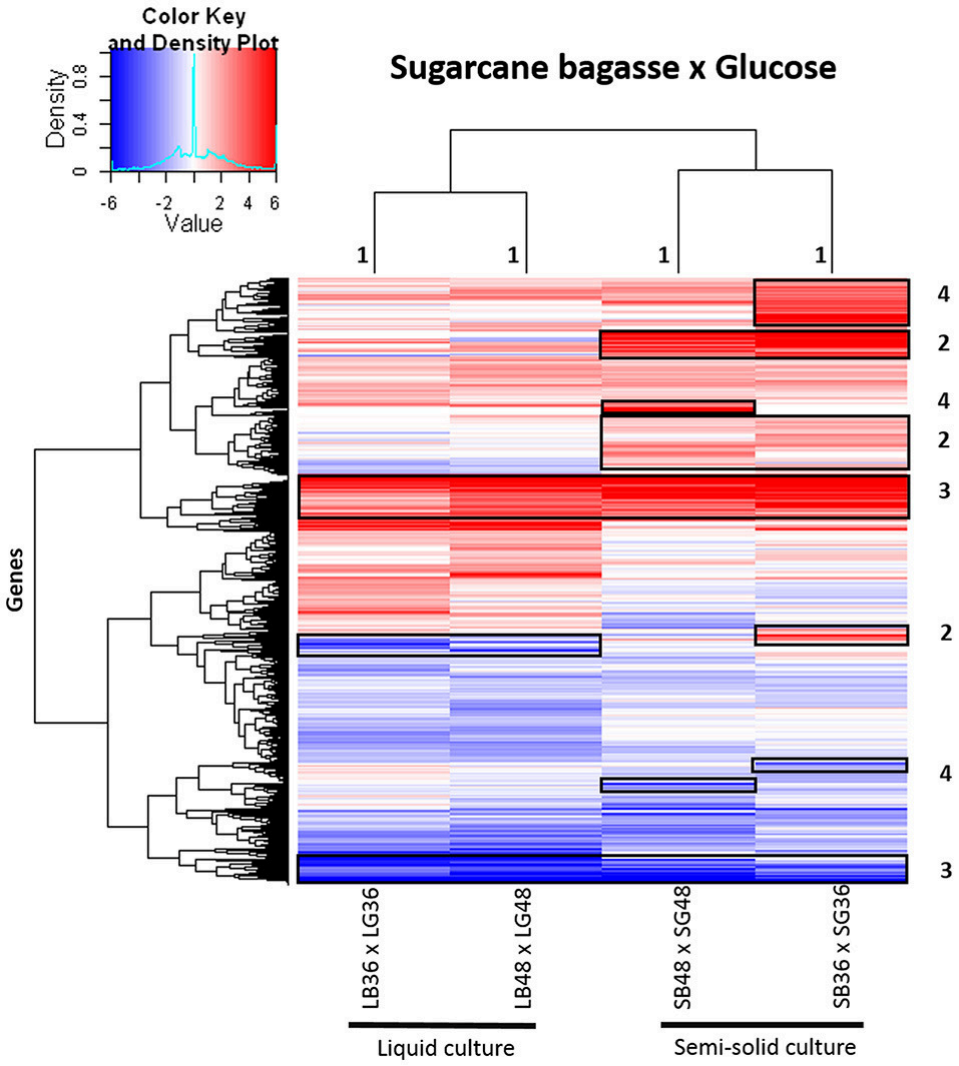
Heatmap of hierarchical clustering of gene transcript modulation patterns observed in *A. tamarii* BLU37 following growth on SB in comparison to glucose as carbon source. Gene transcripts were compared between growth treatments LB36 and LG36, LB48 and LG48, SB36 and SG36, and SB48 and SG48 (padj < 0.01). All log_2_FoldChange values below −6 or above 6 were considered as minimum and maximum values. Categorized groupings of expressed genes one to four are referred to in the main text of the results.

A global heatmap representation of gene expression profiles in *A. tamarii* BLU37 following growth on SB in comparison to glucose revealed considerable modulation of gene expression following growth on this source of plant cell wall polysaccharides (Figure 3). Modulated genes on SB, in comparison to glucose, that grouped exclusively according to culture format (liquid or semi-solid culture), and independent of the growth period, can be observed in Figure 3, region 1. Modulated genes after growth on SB were also identified that grouped according to both culture format and growth period (Figure 3, region 2), according to SB as carbon source, independent of culture format or growth period (Figure 3, region 3), or exclusively according to growth period (Figure 3, region 4). Comparison of expression levels in the predicted genes identified in each of the treatments, whether in growth media supplemented with SB or glucose, revealed that gene expression in *A. tamarii* can also be influenced exclusively according to growth period or culture format, regardless of carbon source (Supplementary Figure 1).

Numerous genes were detected with significant expression fold change on SB as sole carbon source in comparison to glucose at an equivalent growth time point. For LB36, a total of 621 DEGs were detected with significant expression up-regulation, plus a total of 919 DEGs showing down-regulation. For LB48, numbers were greater, with 1187 DEGs up-regulated and 1229 down-regulated. The overall number of DEGs observed following growth on semi-solid media was generally lower than observed on liquid media. For SB36, a total of 755 DEGs were significantly up-regulated, with 449 down-regulated. Similarly for SB48, a total of 914 DEGs were detected as up-regulated and 445 as down-regulated.

#### Gene ontology enrichment analysis of differentially expressed genes

**Figure 4:**
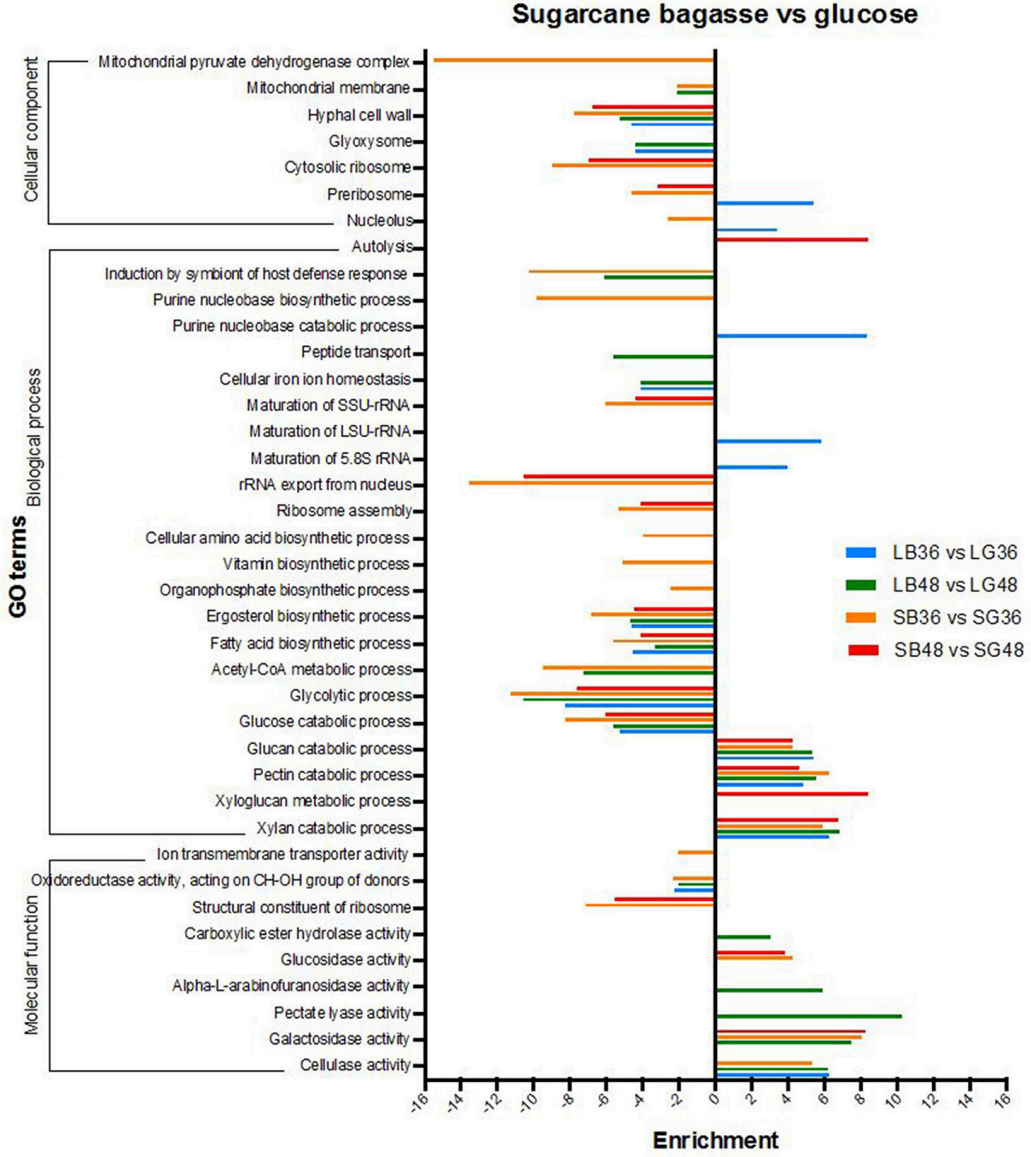
Gene ontology (GO) enrichment analysis of differentially expressed genes in *A. tamarii* BLU37 following growth on steam-exploded sugarcane bagasse (SB) compared with glucose-supplemented treatments. Parameters comprising a cut-off False Discovery Rate (FDR) of 0.0%, a corrected *P*-value of < 0.05 and enrichment of >2 and < −2 were employed for consideration of DEGs between the compared growth treatments.

Differentially expressed genes were analyzed for over- and under-representation according to gene function classification within gene ontology categories (GO) (Figure 4). The majority of annotations for DEGs were classified in biological process subcategories, followed by molecular function and cellular component. Biological process GO terms enriched in DEGs up-regulated following SB hydrolysis included those involved in xylan (GO:0045493), pectin (GO:0045490) and glucan (GO:0009251) catabolic process, with enrichment of up to 4.2 times in all treatments supplemented with SB. Xyloglucan metabolic process (GO:0010411) was the most pronounced of such GO terms, with an enrichment of 8.4 times for the treatment SB48. By contrast, glycolytic process (GO:0006096), glucose catabolic process (GO:0006007), fatty acid (GO:0006633) and ergosterol biosynthetic process (GO:0006696) were abundant terms for down-regulated DEGs following growth in SB-supplemented treatments, indicating that genes classified under these GO terms are more highly expressed in the treatments supplemented with glucose. With regard to molecular function, subcategories cellulase (GO:0008810), galactosidase (GO:0015925), pectate lyase (GO:0030570), alpha-L-arabinofuranosidase (GO:0046556), carboxylic ester hydrolase (GO:0052689) and glucosidase activity (GO:0015926) were all enriched in up-regulated DEGs following growth on SB-supplemented treatments, providing further evidence for *A. tamarii* as a promising fungal species for secretion of cellulases and hemicellulases.

#### CAZyme-encoding genes

**Figure 5:**
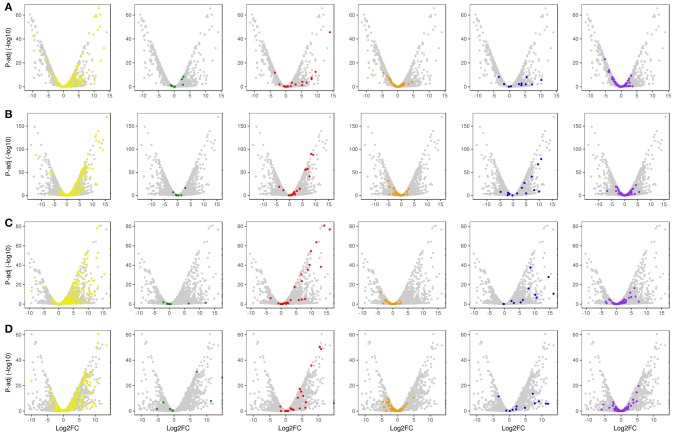
Global volcano scatter plot analysis of expression modulation of CAZyme-encoding genes, transcription factors and un-annotated genes. **(A)** LB36 vs. LG36; **(B)** LB48 vs. LG48; **(C)** SB36 vs. SG36; **(D)** SB48 vs. SG48. Yellow dots, GH family CAZyme-encoding genes; Green dots, CMB family CAZyme-encoding genes; Red dots, CE family CAZyme-encoding genes; Orange dots, GT family CAZyme-encoding genes; Blue dots, PL family CAZyme-encoding genes; Purple dots, Transcription Factor-encoding genes; Gray dots, un-annotated genes.

**Figure 6:**
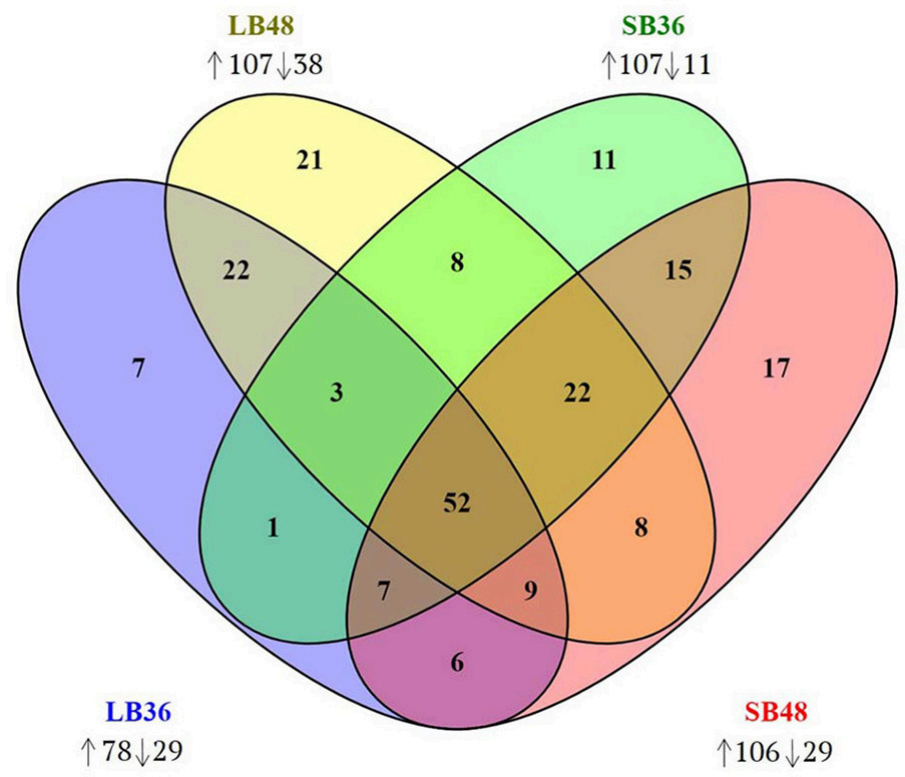
Venn diagram of CAZyme-encoding genes observed in treatments LB36, LB48, SB36, and SB48. Differentially expressed genes were considered significant if relative expression in comparison to equivalent treatments with glucose as carbon source showed at least a 2-fold change and considering a false discovery rate (FDR)-adjusted *P*-value (padj) at 0.01. Arrows pointing up or down: CAZyme-encoding genes up-regulated or down-regulated in each treatment.

With regard to plant cell wall degradation in SB, global analysis of gene expression following fungal growth in the four treatments LB36, LB48, SB36, and SB48 revealed the presence of a total of 311, 314, 337, and 324 expressed CAZyme-encoding genes, respectively, after growth in treatments with this complex carbon source. Differential expression modulation in relation to equivalent treatments with glucose is summarized in Figure 5. *A. tamarii* BLU37 clearly expressed important genes related to SB degradation, with a total of 209 CAZy-encoding genes with statistically significant differential expression on SB, either in liquid or semi-solid treatments, in comparison to equivalent growth treatments with glucose as carbon source (Table 1; Supplementary Table 2). In terms of those with significant modulation of gene expression, genes encoding GH family proteins were the most abundant, with a total of 141 DEGs observed, followed by 15 DEGs encoding CEs, 15 DEGs encoding PLs, four DEGs encoding AA proteins and 32 DEGs encoding GTs. A Venn representation of total numbers of CAZyme-encoding DEGs in treatments LB36, LB48, SB36 and SB48 revealed 50 genes that were common to all four treatments and likely representing those essential in hydrolysis of the complex carbon source SB (Figure 6).

**Table 1:** CAZyme-encoding genes with significant increased expression after growth in treatments with steam exploded bagasse, in comparison with glucose.

					**log**_**2**_**FoldChange** > **2; padj** < **0.01**			
**Class**	**Enzyme description**	**Gene name**	**CAZy Family**	**Gene ID AspGD**	**LB36**	**LB48**	**SB36**	**SB48**
Cellulases	β-1,4-endoglucanase	*celA*	GH12	AO090026000102	11.99	14.21	10.73	9.49
		*celB*	GH7	AO090010000314	10.58	11.78	9.52	7.91
		*eglA*	GH5	AO090005001553	7.64	9.26	10.50	6.53
	cellobiohydroases	*celC*	GH7	AO090001000348	10.16	11.41	10.57	8.31
		*celD*	GH7	AO090012000941	8.41	10.87	9.14	7.35
		*cbhC*	GH6	AO090038000439	11.16	12.20	10.63	7.96
	β-glycosidase	*bgl3*	GH1	AO090003000497	5.35	7.24	6.19	4.15
		*bglD*	GH3	AO090701000274	5.21	5.55	8.57	6.66
		*bgl5*	GH3	AO090001000544	4.32	6.00	6.09	5.27
		–	GH3	AO090701000244	4.00	5.85	5.32	3.98
		–	GH3	AO090005000337	6.61	7.29	8.08	7.56
	–	Ortholog *A. fumigatus exg3*	GH5	AO090005000423	5.01	7.05	8.82	6.02
	Lytic Polysaccharide Mono-Oxygenase (LPMO)	–	AA9	AO090023000787	10.51	11.12	8.01	7.32
		–	AA9	AO090023000159	7.07	6.70	6.70	4.11
		–	AA9 CBM1	AO090103000087	5.52	6.16	5.62	3.71
Hemicellulases	endo-β-1,3(4)-glucanase	Ortholog *A. fumigatus eng3*	GH16	AO090003000825	3.03	3.64	5.19	6.78
	endoxylanase	*xynG1*	GH11	AO090001000111	10.68	11.47	9.66	6.84
	xylanase	*xynG2*	GH11	AO090120000026	13.19	15.55	15.41	10.98
	endo-β-1,4-xylanase	*XynF1*	GH10	AO090103000423	12.18	14.05	12.99	10.43
	–	Ortholog *A. niger xynD*	GH43	AO090003000239	4.26	5.15	7.07	6.01
	–	Ortholog *A. niger xynD*	GH43	AO090026000804	2.58	4.25	3.61	6.46
	β-xylosidase	*xylA*	GH3	AO090005000986	4.90	5.29	12.11	10.50
		*xylB*	GH43	AO090005000698	5.75	6.26	9.00	6.70
	α-xylosidase	–	GH31	AO090005000768	4.74	7.63	9.53	8.43
				AO090005000767	5.35	6.99	9.69	8.39
	xyloglucan endo-β-1,4-glucanase	Ortholog *A. oryzae celA*	GH12	AO090003000905	5.67	6.19	4.85	2.71
	acetyl xylan esterase	*axeA*	CE1	AO090011000745	8.19	8.27	14.29	10.38
	β-galactosidase	*bglB*	GH3	AO090012000135	3.17	6.74	12.31	13.55
	α-galactosidase	Ortholog *A. nidulans aglE*	GH36	AO090011000063	4.32	5.93	8.36	4.36
	arabinogalactan endo-1,4-β- galactosidase	Ortholog *A. nidulans galA*	GH53	AO090001000492	2.19	4.44	7.78	5.65
	arabinofuranosidase	–	GH43	AO090701000886	9.71	10.17	12.74	8.79
	α-L-arabinofuranosidase	Ortholog *A. nidulans axhB*	GH62	AO090701000885	11.50	12.68	13.49	10.81
	α-L-fucosidase	Ortholog *A. nidulans afcA*	GH95	AO090005000382	4.86	8.83	10.74	7.06
	α-glucuronidase	Ortholog *A. nidulans aguA*	GH67	AO090026000127	5.85	7.93	9.46	6.55
	β-N-acetylhexosaminidase	–	GH20	AO090701000314	3.25	4.83	4.82	4.77
	feruloyl esterase	Ortholog *A. nidulans faeC*	CE1	AO090701000884	14.03	–	16.13	10.83
	feruloyl esterase	Ortholog *A. nidulans faeC*	CE1	AO090023000158	9.44	9.07	9.89	7.83
	acetylmannan esterase	*ameA*	CE16	AO090005001552	6.60	6.04	6.85	4.31
Pectinases	pectin lyase	Ortholog *A. oryzae pel1*	PL1	AO090010000087	5.19	9.60	14.55	11.24
	pectate lyase	Ortholog *A. nidulans pelA*	PL1	AO090011000673	10.17	10.78	16.19	11.89
	–	Ortholog *A. nidulans plyC*	PL1	AO090102000072	5.48	7.15	10.15	7.20
	–	–	PL9	AO090038000131	3.85	8.12	8.57	9.10
	polygalacturonase	Ortholog *A. nidulans xghA*	GH28	AO090102000011	10.10	–	13.24	9.49
	pectinesterase	Ortholog *A. nidulans pmeA*	CE8	AO090102000010	8.16	7.66	13.19	–
Others	α-amylase	–	GH13	AO090103000378	4.65	4.74	6.00	4.98
	Extracellular lipase (cutinase)	*cutL*	CE5	AO090005000029	5.23	6.20	6.70	4.69

DEGs involved in cellulose depolymerization included β-1,4-endoglucanases, cellobiohydrolases, β-glucosidases, and lytic polysaccharide mono-oxygenases (LPMO). Genes with expression modulation of up to 10-fold were also observed amongst the DEGs, including numerous encoding hemicellulases related to xylan depolymerization such as xylanase, endoxylanase, endo-β-1,4-xylanase, α-L-arabinofuranosidase, arabinofuranosidase, and feruloyl esterase. Genes related to pectin depolymerization were also highly expressed, such as for those encoding polygalacturonase, pectinesterase, pectin and pectate lyase. A total of 40 CAZyme-encoding DEGs were exclusive to specific treatments with sugarcane. Interestingly, most were expressed positively in the treatment SB36 and were related to pectin hydrolysis. These included, for example, orthologos for genes encoding a polygalacturonase (AO090009000470), a rhamnogalacturonan hydrolase (AO090102000139), a pectin lyase (AO090010000030), and a β-glucosidase (AO090701000841).

#### Differentially expressed transcription factor and transporter-encoding genes

A total of 80 transcription factor genes were differentially expressed in *A. tamarii* BLU37 following growth on sugarcane in comparison with equivalent treatments with glucose (padj < 0.01) (Supplementary Table 3). These comprised genes with transcription factor activity domains in families such as Zn2/Cys6 DNA-binding domain, Homeodomain, C2H2 zinc fingers, helix-loop-helix DNA-binding domain, GATA zinc finger and Basic-leucine zipper (bZIP). An ortholog for the transcription factor gene XlnR (AO090012000267), known to be responsible for activation of genes involved in xylan and cellulose degradation, showed an increased expression of up to 1-fold in LB48 and 1.46-fold in SB36. Additionally, the XlnR gene ortholog AO090003001292, showed increased expression of 2.06-fold in LB48 and 1.75-fold in SB48. Similarly, an ortholog of the ClrA transcription factor gene AO090011000944, which is positively regulated by XlnR, was differentially modulated 2.33-fold in LB36, 2.34-fold in LB48 and 2.07-fold in SB36. Two transcription factors controlling pectinase-encoding genes, namely RhaR (AO090005000121) and AraR (AO090003001292), were also up-regulated, 1.80-fold in LB48, 2.60-fold in SB36, 2.05-fold in LB48 and 1.74 in SB48. Interestingly, an ortholog of the BlrA gene (AO090005001041), a DNA binding transcription factor involved in asexual sporulation in fungi, displayed an early up-regulation in the treatments on sugarcane bagasse, with a 1.11-fold increase in expression in LB36 followed by a 0.73-fold decrease in LB48, together with a 7.40-fold increase in expression in SB36 and subsequent 1.70-fold decrease in gene expression in SB48. Transcription factor genes that are known to act in repression of cellulases and hemicellulases, such as the zinc-finger carbon catabolite repressor transcription factor CreA (AO090026000464) (Ruijter and Visser, 1997), together with Ace1 (AO090005001502) and Ace2 (AO090003000678), whilst observed amongst the unigenes identified in the *A. tamarii* transcriptome, were not significantly differentially expressed on sugarcane bagasse in comparison to growth on glucose as carbon source.

A total of 155 transporter genes were differentially expressed across the four treatments on sugarcane, in comparison to glucose (Supplementary Table 4). Highly expressed genes related to sugar transport were, in the majority, those known to be positively regulated by the transcription factor XlnR and coding for Major Facilitator Superfamily (MFS) proteins. Orthologs of the genes AO090003000782, AO090001000069, and AO090003001277 were the most positively differentially expressed of such genes in all treatments on sugarcane, in comparison to glucose. The putative D-xylose transmembrane transporter XtrD (AO090001000069) was also highly expressed on sugarcane bagasse (log2FC 5.02 in LB36, 5.57 in LB48, 7.16 in SB36 and 4.68 in SB48), as was the putative cellobiose transporter cdt-2 gene ortholog (AO090003001277), expressed at log2FC levels 6.53 in LB36, 9.10 in LB48, 8.48 in SB36 and 6.07 in SB48.

#### Validation of RNAseq analysis by RT-qPCR

**Figure 7:**
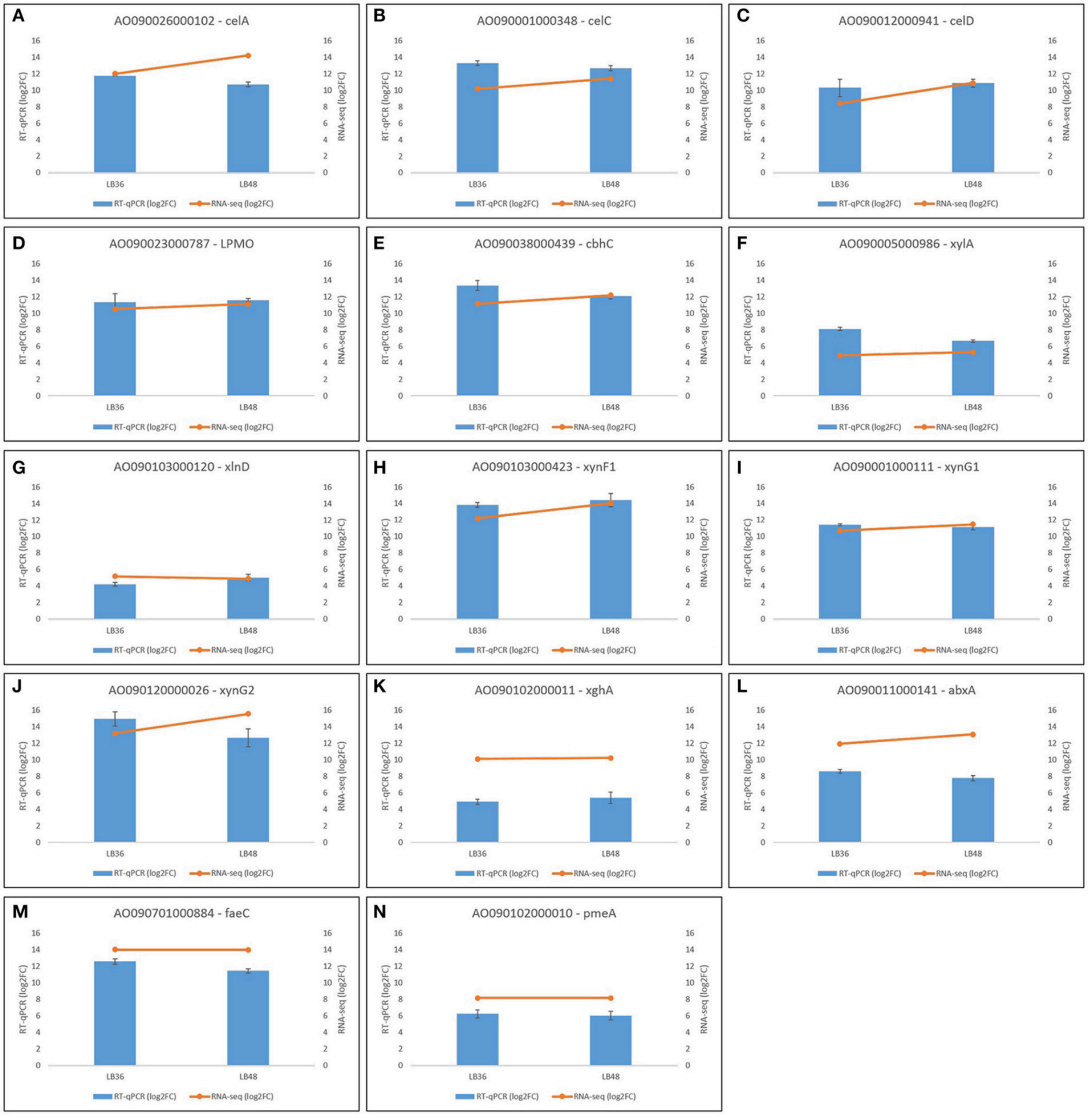
Validation of differential gene expression profiles based on RNAseq through RT-qPCR analysis in selected genes **(A–N)** in *Aspergillus tamarii* BLU37. Black bars represent standard error values, which are based on analysis of three biological replicates per treatment and three technical amplification replicates.

In order to validate Illumina RNAseq-derived gene expression data, a total of 14 highly expressed CAZyme-encoding genes were selected for expression profile analysis by RT-qPCR. These gene orthologs, which are known to be positively regulated by XlnR, comprised cellobiohydrolases (AO090001000348, AO090012000941, AO090038000439), endoxylanases (AO090103000423, AO090120000026, AO090001000111), feruloyl esterase (AO090701000884), pectinesterase (AO090102000010), polygalacturonase (AO090102000011), arabinofuranosidase (AO090103000120), β-xylosidase (AO090005000986), exoarabinase (AO090011000141), endoglucanase (AO090026000102), and a Lytic Polysaccharide Mono-Oxygenase (LPMO) (AO090023000787). Both RNAseq and RT-qPCR gene expression values are represented in log2FoldChange, showing broad agreement in expression pattern tendencies to up- or down-regulation in each of the four treatment comparisons (Figure 7). Primer sequences for target genes selected for validation via RT-qPCR are available in Supplementary Table 5.

## Discussion

Efficient 2G bioethanol production systems require the complete hydrolysis of hexose and pentose sugars present in plant biomass. In this study, a global analysis of the transcriptome revealed considerable ligninolytic potential in the ascomycete fungus *A. tamarii* BLU37. With an abundance of expressed CAZyme-encoding genes, data on the transcriptional response to contrasting carbon sources of sugarcane bagasse and glucose both increase understanding of the mechanisms involved in enzyme secretion and efficiency, and serve as a resource for exploitation of genes encoding transcription factors, transporters and enzymes involved in the degradation of plant biomass for engineering of improved microorganisms.

As plant pathogens or saprophytes, many filamentous fungi are efficient in hydrolytic extracellular enzyme secretion for plant biomass depolymerization. A number of species belonging to the genus *Aspergillus* are efficient in secretion of a large repertoire of glycosyl hydrolases appropriate for industrial application in lignocellulosic biomass degradation (Machida et al., 2005; Duarte et al., 2012; Jaramillo et al., 2013; Pirota et al., 2014). The strain examined in this study was originally isolated from composting cotton textile waste material (Siqueira et al., 2009). Typically, this species is associated with plant biomass degradation as a spoilage fungus, with reports of isolation from food and feed products (Rodrigues et al., 2011; Midorikawa et al., 2014; Martins et al., 2017; Prencipe et al., 2018). Previous studies have also highlighted the potential in this species in depolymerization of xylan, through secretion of β-1,4-endoxylanases (Gouda and Abdel-Naby, 2002; da Silva et al., 2014). Efficient secretion of such xylanases has also been reported in the strain *A. tamarii* BLU37 (Duarte et al., 2012; Monclaro et al., 2016). Whilst the transcriptome in different Aspergillus species during degradation of plant biomass has been the subject of recent investigation (de Souza et al., 2011; Delmas et al., 2012; Pullan et al., 2014; van Munster et al., 2014; Miao et al., 2015; Borin et al., 2017), despite the clear potential of *A. tamarii* as a xylanolytic species, there has been no investigation, prior to the current study, of the transcriptome of this species during saccharification of plant biomass.

The *A. tamarii* BLU37 transcriptome in response to SB was mapped to the annotated reference *A. oryzae* RIB40 genome, with the total number of expressed genes detected (7126 genes) similar to that previously observed in *A. niger* (7359 genes) when grown on this same lignocellulosic substrate as sole carbon source (Borin et al., 2017). Global analysis of gene expression revealed DEGs not only induced or repressed according to culture treatments with SB or C, but also according to semi-solid versus liquid culture (Supplementary Figure 1). Culture format has previously also been reported to influence enzyme secretion in *Aspergillus* species, with greater protein production in *A. oryzae* following solid-state fermentation (Wang et al., 2010b). Similarly, in the case of *A. niger*, solid-state fermentation of pretreated sugarcane bagasse was reported to favor endoglucanase and xylanase secretion, with submerged fermentation favoring β-glucosidase production (Vasconcellos et al., 2015).

Enzymes involved in the degradation of plant cell wall polysaccharides are distributed in different classes of glycosidases based on their primary amino acid sequence and related catalytic modules. Currently, these enzymes are classified as carbohydrate esterases (CEs), polysaccharide lyases (PLs), glycoside hydrolases (GHs), glycosyltransferases (GTs), as enzymes with the auxiliary activities (AAs), and as carbohydrate- binding modules (CBMs). As lignocellulose deconstruction requires the activities of a large number of these CAZymes, analysis of DEGs included a comparison of CAZyme-encoding genes. Considering that *Aspergillus* sp. are able to colonize a variety of plant biomass polysaccharides, and given that sugarcane bagasse has been shown to comprise approximately 35% cellulose, 24% hemicellulose and 22% lignin (Rezende et al., 2011), it was expected that *A. tamarii* BLU37 would secrete many enzymes from different CAZy families when growing on SB as carbon source. The majority of the CAZyme-encoding genes observed amongst DEGs on SB were those encoding proteins classified across 36 GH families. The total of 141 GH family DEGs reflects the similar numbers identified in the genomes of *A. oryzae, A. terreus, A. niger* and *A. nidulans*, with respective totals of 194, 186, 157, and 172 predicted GH family CAZyme-encoding genes. Greatest numbers of genes with differential expression on SB included those from GH families 3, 5, 31, 43, and 92. CAZyme-encoding DEGs classified in CE families (15 genes) and PL families (15 genes) were also similar in numbers to those observed in the *A. oryzae* genome, where 14 CE and 20 PL genes have been annotated (Benoit et al., 2015). DEGs encoding GTs were generally less expressed on SB in comparison to glucose, indicating a probable lack of involvement in degradation of this biomass. GTs represent a diverse family of enzymes that function in the cell in many activities relating to structure, storage and signaling. Numerous GTs can transfer sugar residues from an activated sugar donor residue to specific acceptor molecules, resulting in the formation of glycosidic bonds. As such, this family of enzymes can enable the biosynthesis of an infinite number of oligosaccharides, polysaccharides and glycoconjugates (Taniguchi et al., 2002; Coutinho et al., 2003). As such, in contrast to sugarcane bagasse, where enzyme activity is involved in the degradation of plant cell wall polysaccharides, an increased expression of GTs on glucose may be expected, likely resulting in the biosynthesis of oligosaccharides and polysaccharides required for fungal metabolism. Such observations have also been reported in *A. fumigatus* on different lignocellulosic biomass sources (Miao et al., 2015; de Gouvêa et al., 2018), with many such GTs involved in the biosynthesis of fungal cell wall chitins and ergosterol glycosylation (Klutts et al., 2006; Castell-Miller et al., 2016). Additional DEGs likely to be not involved in the plant cell wall degradation included six GH18 putative chitinases, with activity likely associated with cell wall dynamics during fungal growth, as also observed in the ascomycete fungus *Malbranchea cinnamomea* (Hüttner et al., 2017).

Genes related to cellulose and hemicellulose deconstruction were highly expressed during liquid and semi-solid culture on SB (Supplementary Table 2), including cellobiohydrolases (CBHs) (celC - AO090001000348, celD - AO090012000941 and chbC - AO090038000439), endoglucanases (EGs) (celA - AO090026000102, celB - AO090010000314 and eglA - AO090005001553), and β-glucosidases (BGLs) (bgl3 - AO090003000497, bglD - AO090701000274 and bgl5 - AO090001000544). Two putative β-glucosidases (AO090701000244 and AO090005000337) and one putative exoglucanase (AO090005000423) were also observed with high levels of expression during liquid and semi-solid culture. In the case of the CAZy family AA9, classified as a member of the copper-dependent lytic polysaccharide monooxygenases (LPMOs) and responsible for cellulose cleavage through an oxidative reaction, two AA9 genes (AO090023000787 and AO090023000159) and two AA9 genes linked by a carbohydrate-binding module (CBM1) (AO090103000087 and AO090005000531) were observed amongst the highly expressed DEGs on SB, as also reported in *A. niger* (Borin et al., 2017). Cellulose-binding CBMs are frequently observed amongst fungal enzymes, playing roles in anchoring enzymes to crystalline cellulose (Igarashi et al., 2009), conferring enzyme and substrate specificity and enhancing enzyme activity (Crouch et al., 2016). CBM1s have been estimated to represent over 30% of CBMs in Ascomycete and Basidiomycetes (Várnai et al., 2014).

Whilst sugarcane bagasse hemicellulose and pectin have structures composed of xylan, galactan and arabinan polymers, many of the highly expressed DEGs from liquid and semi-solid cultures grown on sugarcane bagasse were related to xylan saccharification, corroborating enzyme activity data and previous studies on *A. tamarii*. The endoxylanases (xynG1 - AO090001000111, xynF1 - AO090103000423) and xylanase (xynG2 - AO090120000026), for example, were up-regulated more than 10-fold in SB. Xylosidases involved in xylooligomer hydrolysis into xylose, such as α-xylosidases (AO090005000768 and AO090005000767) and β-xylosidases (xylA - AO090005000986 and xylB - AO090005000698), also showed high expression levels.

Genes that encode accessory enzymes involved in galactan breakdown, such as β-galactosidase and α-galactosidase (AO090012000135 and AO090011000063), as well as those involved in xylan side chain removal, such as arabinofuranosidases (AO090701000886 and AO090701000885) and feruloyl esterases (AO090701000884 and AO090023000158), were all also highly expressed in liquid and semi-solid SB culture.

Fungi from the genus *Aspergillus* possess a variety of genes encoding enzymes related to pectin degradation. *A. niger* possesses over 60 such genes and *A. oryzae* over 90 genes encoding for pectinolytic enzymes (Coutinho et al., 2009; Martens-Uzunova and Schaap, 2009). In our study, we detected numerous pectin-related DEGs with increased expression on SB. Genes with increased expression on semi-solid SB included a number from the PL1 family (AO090010000087, AO090011000673, and AO090102000072), the PL9 family (AO090038000131), pectinase-encoding genes from the GH28 family (exopolygalacturonase - AO090005001400; polygalacturonase - AO090009000470 and AO090138000086; and rhamnogalacturonan hydrolase - AO090102000139), as well as one gene from the PL1 family, a pectin lyase (AO090010000030). In contrast, a number of PL1 family DEGs displayed increased expression exclusively in liquid SB culture, namely AO090012000451, AO090701000321, and AO090010000706. Such data reveal that culture format of this carbon source, in addition to cultivation time, can influence gene expression. Although pectin is less abundant in sugarcane bagasse in comparison to xylan, in terms of chemical composition (de Souza et al., 2013), the presence of PL family members amongst the DEGs also indicates pectin breakdown in SB. Similar increased expression has previously been observed in PL4 family member genes in *A. niger* and *A. fumigatus* grown on SB (Borin et al., 2017; de Gouvêa et al., 2018).

Transcription factors perform major roles in the regulation of gene expression. Our data revealed a total of 80 differentially expressed transcription factor genes, from the families Zn2/Cys6 DNA-binding domain, Homeodomain-like, C2H2 zinc fingers, helix-loop-helix DNA-binding domain, GATA zinc finger and Basic-leucine zipper (bZIP) transcription factor. Amongst the DEGs, a total of 42 transcription factors were identified, from Zn clusters, a family commonly found across fungal species (Shelest, 2017). Of these, the transcription factor XlnR is known to play an important role in xylanolytic transcriptional activation in fungi (van Peij et al., 1998), controlling expression of a wide range of genes, including those encoding xylanases and enzymes in the D-xylose metabolic pathway (Hasper et al., 2000; de Groot et al., 2007). This TF is also involved in regulation of expression of genes encoding endocellulases (Gielkens et al., 1999). In the case of *Aspergilli* specifically, XlnR is known, in the presence of D-xylose, to induce xylanases, β-xylosidases, cellobiohydrolases, endoglucanases, galactosidases, arabinofuranosidases and carbohydrate esterases (Mach-Aigner et al., 2012). In this study, XlnR was up-regulated according to SB substrate, as observed previously in *A. niger* in response to SB, over a similar time course (Borin et al., 2017), as well as in *A. fumigatus* on rice straw (Miao et al., 2015). Up-regulation was also observed in the ClrA gene following growth in LB36 and LB48. Together with XlnR, this gene is also known to act positively in cellulase production in *A. niger* (Raulo et al., 2016). Two transcription factors controlling pectinase-encoding genes, namely RhaR and AraR were also up-regulated in LB and SB. The major pectinase-encoding genes in *A. niger* have been shown to be under the control of the transcription factors RhaR, AraR, and GaaR (Kowalczyk et al., 2017), suggesting that the up-regulation of the RhaR and AraR observed in our study, also indicates a role in induction of pectinase-encoding gene expression in *A. tamarii*, releasing lignin associated with pectin in the sugarcane bagasse cell wall.

Following the breakdown of lignocellulosic biomass into mono- and disaccharide sugars, transport into the cell will involve numerous sugar transporters. Although genome sequence data has revealed that filamentous fungi indeed harbor many genes encoding sugar transporters, relatively few have been functionally validated to date (dos Reis et al., 2016). MFS transporter proteins are known to transport small soluble molecules such as sugars across ion gradients (Pao et al., 1998). Following comparison with all predicted MFS proteins in the annotated reference genome sequences for *A. oryzae* (508), *A. nidulans* (357), and *A. fumigatus* (278) (Ferreira et al., 2005), our data revealed over 150 potential transporter genes differentially expressed in *A. tamarii* following growth on sugarcane bagasse in comparison to glucose. The relatively large number of transporters identified in the genus Aspergillus, in comparison with *T. reesei* and *N. crassa*, may explain their greater ability to transport small solutes (Miao et al., 2015). Previously, de Souza et al. (2011) characterized seven genes encoding predicted transporters in *A. niger*, with increased expression in response to sugarcane bagasse and repression in the presence of glucose, suggesting a role in xylose transport. From our data, numerous differentially expressed transporter genes potentially involved in SB degradation were identified. Increased expression of the putative D-xylose transmembrane transporter gene *xtrD*, for example, likely indicates involvement in transport into the cell of free D-xylose released from xylan following activity of highly expressed xylanases, endoxylanases and xylosidases. In accord with our findings, the *xtrD* gene from *A. nidulans*, whilst able to accept multiple sugars (xylose, glucose, galactose, and mannose), shows high affinity for xylose, being induced by xylose in an XlnR-dependent manner, and repressed by glucose in a CreA-dependent manner (Colabardini et al., 2014). Similarly, significant increased expression of the *cdt-2* gene ortholog, a putative cellobiose transporter, was observed across all four treatments with SB as carbon source, likely associated with cellobiose transport into the fungal cell. Given the inability of *S. cerevisiae* to transport sugars other than glucose (Young et al., 2010), the identification of transporters for pentose sugars and cellodextrins in *A. tamarii* will offer potential in genetic modification of Saccharomyces yeasts, facilitating transport and fermentation of D-xylose.

Whilst there is evidence for conservation of the genomic potential for plant biomass degradation across certain *Aspergillus* species (Benoit et al., 2015), investigation into gene expression and enzyme secretion has shown considerable variation across species and during cultivation on different carbon sources. This first analysis of the repertoire of CAZyme-, transcription factor- and sugar transporter-encoding genes in *A. tamarii* modulated in response to SB increases our understanding of enzymatic saccharification of this lignocellulosic biomass. Transcriptome data serves as a resource for economically viable biorefinary applications, with potential for application in improvement of enzymatic conversion of biomass to value-added products, through genetic improvement of both lignocellulolytic filamentous fungi, as well as yeasts employed in the fermentation of hexose and pentose sugars in hydrolysates in industrial 2G ethanol production.

## Data availability

The Illumina RNAseq datasets generated for this study can be found in the NCBI Sequence Read Archive (SRA) database (BioProject ID PRJNA479954, SRA accession: SRP152413).

## Author contributions

RM, EN, and EF planned the experiments. GM and CC performed the bioassays, enzyme analyses, RNA and cDNA preparation, and sequence data analysis. RT, MC, OS, and PG participated in sequence data analysis and editing of the manuscript. RM conceived the study, participated in bioassays, RNA preparation for cDNA library construction, sequence data analysis, and drafted the manuscript. All authors have contributed to, read and approved the final version of the manuscript.

### Conflict of interest statement

The authors declare that the research was conducted in the absence of any commercial or financial relationships that could be construed as a potential conflict of interest.
