# Supplementary material for: Analysis of the Transcriptome in Aspergillus tamarii During Enzymatic Degradation of Sugarcane Bagasse
Source: Front Bioeng Biotechnol. 2018 Sep 18;6:123. doi: 10.3389/fbioe.2018.00123 (PMC6153317; doi:10.3389/fbioe.2018.00123)
Supplement: Supplementary file 1 [file Table_1.DOCX]

**Supplementary Table 1:** Summary of Illumina RNAseq data statistics for each *Aspergillus tamarii* BLU37 cDNA library

|  | **Before adapter trimming** | | | | **After adapter trimming** | | | |
| --- | --- | --- | --- | --- | --- | --- | --- | --- |
| **Sample Code** | **Yield (Gbases) per library** | **Sequence reads (Million)** | **Percent reads with quality Fastq QC>30** | **Mean quality Fastq QC>30 score** | **Yield (Gbases) per library** | **Reads (Million)** | **Percent reads with quality Fastq QC>30** | **Mean quality Fastq QC>30 score** |
| LB361 | 6.58 | 65.8 | 79.58 | 32.17 | 5.46 | 63.61 | 80.77 | 34.94 |
| LB362 | 5.46 | 54.6 | 79.05 | 32.02 | 4.52 | 52.69 | 80.59 | 34.86 |
| LB481 | 5.13 | 51.3 | 79.58 | 32.17 | 4.26 | 49.59 | 80.68 | 34.90 |
| LB482 | 5.19 | 51.9 | 79.66 | 32.19 | 4.31 | 50.16 | 80.75 | 34.93 |
| SB361 | 4.78 | 47.8 | 79.8 | 32.24 | 3.98 | 46.24 | 80.73 | 34.89 |
| SB362 | 4.56 | 45.6 | 79.49 | 32.14 | 3.79 | 44.09 | 80.63 | 34.86 |
| SB481 | 5.35 | 53.5 | 79.12 | 32.04 | 4.42 | 51.51 | 80.38 | 34.81 |
| SB482 | 5.07 | 50.7 | 78.96 | 32.01 | 4.18 | 48.80 | 80.30 | 34.75 |
| LG361 | 5.11 | 51.1 | 77.21 | 31.46 | 4.15 | 48.73 | 80.19 | 34.60 |
| LG362 | 5.18 | 51.8 | 77.63 | 31.58 | 4.22 | 49.50 | 80.34 | 34.67 |
| LG481 | 5.45 | 54.5 | 77.31 | 31.48 | 4.42 | 51.92 | 80.39 | 34.76 |
| LG482 | 5.61 | 56.1 | 77.57 | 31.57 | 4.57 | 53.57 | 80.23 | 34.61 |
| SG361 | 4.38 | 43.8 | 77.17 | 31.44 | 3.56 | 41.73 | 80.07 | 34.56 |
| SG362 | 5.18 | 51.8 | 76.86 | 31.36 | 4.20 | 49.41 | 79.92 | 34.53 |
| SG481 | 4.79 | 47.9 | 74.98 | 30.77 | 3.79 | 45.04 | 79.72 | 34.66 |
| SG482 | 6 | 60 | 76.47 | 31.24 | 4.84 | 56.92 | 79.82 | 34.47 |
