# Supplementary material for: Analysis of the Transcriptome in Aspergillus tamarii During Enzymatic Degradation of Sugarcane Bagasse
Source: Front Bioeng Biotechnol. 2018 Sep 18;6:123. doi: 10.3389/fbioe.2018.00123 (PMC6153317; doi:10.3389/fbioe.2018.00123)
Supplement: Supplementary file 5 [file Table_5.DOCX]

**Supplementary Table 5.** List of target genes and specific primer sequences for qRT-PCR validation of differential gene expression

| **Target Aspergillus / Genbank Gene ID** | **Gene predicted function** | **Forward Primer (5’-3’)** | **Reverse Primer (5’-3’)** |
| --- | --- | --- | --- |
| AO090026000102 | β-1,4-endoglucanase | CATCTCACATCTAAGCACGATTTC | TGACAGTGGCAGTCCATTT |
| AO090001000348 | Cellobiohydrolase | TGGTTTCCAGTGCCTGTATTAG | GTCCACGGGATCTCAAGTTTAT |
| AO090012000941 | Cellobiohydrolase | TCGTCTGCCATCAGGTATAGA | TACGAGGGTACTTACGGTGTTA |
| AO090023000787 | Lytic Polysaccharide Mono-Oxygenase (LPMO) | GAACAGAATACCAGGGTCAGTATC | GAAGGATGGTGCTCAGAACTAC |
| AO090038000439 | Cellobiohydrolase | GAGTACCTGATTAGCGATGGTG | AGATGATCTGAGTGTCCGAGTA |
| AO090005000986 | β-xylosidase | CACTTGAACAACACAGCCTTC | GTTGTATCCTGGTCGGTATGAG |
| AO090103000120 | α-N-arabinofuranosidase/xylan 1,4-β-xylosidase/xylosyltransferase | CTGTGCCTTGAGAGAATCTAACT | CACCCAACTACAGGGAAACTAC |
| AO090103000423 | endo-β-1,4-xylanase | GCATCCTTGGCTTGGTAGTT | GTCAACGCTTGTCTCGATGT |
| AO090001000111 | endoxylanase | GTGGACTAACTGCGACAACT | CCAGCCGTAAAGAGAGACATAG |
| AO090120000026 | xylanase | CGTAGGACTCAACGATGTAGTATTC | CGGCGGAGCAATCTCTTATT |
| AO090102000011 | polygalacturonase | GACGAAGTGGCATCCATAGT | GCGTCCGACCCTACTTTATATC |
| AO090701000884 | feruloyl esterase | CCTCAGCACAACCCGTATAATC | CGTACATTGCGAGACCACTATG |
| AO090011000141 | exoarabinase | GGGCGATGAAACTCCATACTT | CGGACATTCCCAATCCATTCT |
| AO090102000010 | pectinesterase | CTCTGTCCTGCCAGACATTATC | TACTCTCGTCCCGACGTATC |
| *EF422213 | β-tubulin | GAAGGTCTCGTCGGAGTGCTC | AGGTCTCCGACACCGTTGTT |
| *AO090011000414 | GAPDH | GAAGGGGAACCCTCATTCATC | TGGCAATGTAGGCAGTCAGG |

*Gene expression levels normalized against stable *Aspergillus* reference genes β-tubulin (McKelvey & Murphy, 2010) and GAPDH (Wang et al., 2010b)
