# Supplementary material for: Analysis of the Transcriptome in Aspergillus tamarii During Enzymatic Degradation of Sugarcane Bagasse
Source: Front Bioeng Biotechnol. 2018 Sep 18;6:123. doi: 10.3389/fbioe.2018.00123 (PMC6153317; doi:10.3389/fbioe.2018.00123)

SUPPLEMENTARY FIGURE 1. Heatmaps of hierarchical clustering of gene transcript modulation patterns observed in *A. tamarii* BLU37 following growth on SB (A) or glucose (B) as carbon source. Gene transcripts were compared according to time point as well as culture format (padj < 0.01). All log_2FoldChange values below -6 or above 6 were considered as minimum and maximum values.

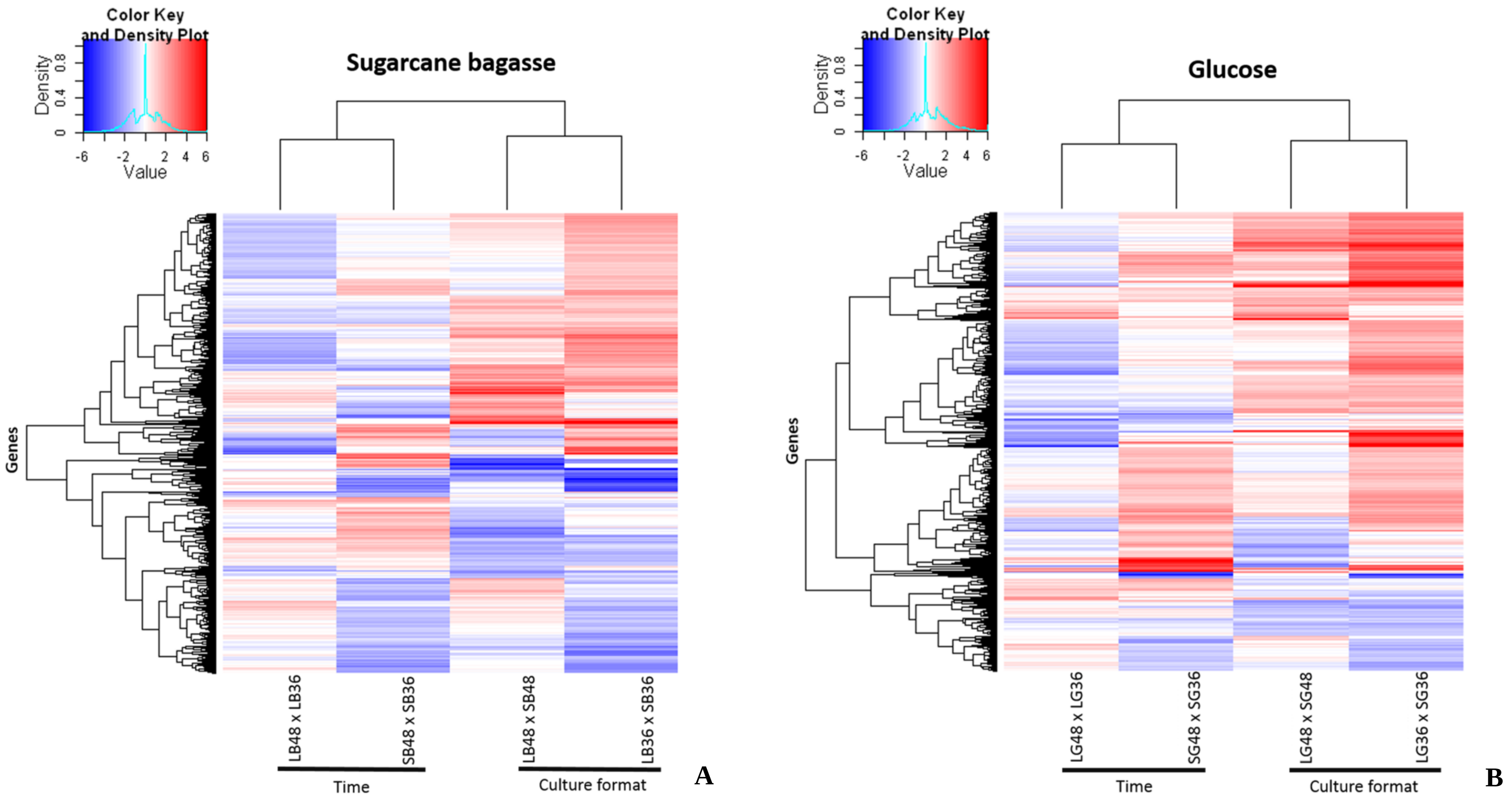

Supplement: Supplementary file 6 [file Image_1.pdf]
